# Patients with Darier disease have an increased risk of keratinocyte carcinoma: a Swedish registry-based nationwide cohort study

**DOI:** 10.1186/s13023-024-03497-z

**Published:** 2024-12-16

**Authors:** Rahime Inci, Martin Gillstedt, Roope A. Kallionpää, Sirkku Peltonen, Sam Polesie

**Affiliations:** 1 Department of Dermatology and Venereology, Institute of Clinical Sciences, Sahlgrenska Academy, University of Gothenburg, Gröna stråket 16, Gothenburg, SE-413 45 Sweden; 2 Region Västra Götaland, Department of Dermatology and Venereology, Sahlgrenska University Hospital, Gothenburg, Sweden; 3 Cancer Research Unit and FICAN West Cancer Centre, Institute of Biomedicine, University of Turku, Turku, Finland; 4 Department of Dermatology and Allergology, University of Helsinki and Helsinki University Hospital, Helsinki, Finland

**Keywords:** Basal cell carcinoma, Cohort investigation, Darier disease, Epidemiology, Melanoma, Observational study, Squamous cell carcinoma

## Abstract

**Background:**

Darier disease is a genodermatosis which manifests as hyperkeratotic papules and superficial erosions mainly in seborrheic skin areas. This retrospective registry-based cohort study aimed to estimate the association between Darier disease and skin cancer.

**Results:**

Patients diagnosed with Darier disease were identified from the patient registry of Sahlgrenska University Hospital (Gothenburg, Sweden) in 2016–2020. The local cohort included 13 patients. Verification of Darier disease diagnosis in the National Patient Registry showed positive predictive value of more than 90%. National Patient Registry was searched for Darier disease in 2001–2020, Swedish Cancer Registry for cancers and Prescribed Drug Register for medications. The national cohort included 770 patients and tenfold matched control cohort. Patients with Darier disease had an increased relative risk of keratinocyte carcinoma (basal cell carcinoma and cutaneous squamous cell carcinoma combined) (hazard ratio [HR], 1.6, 95% confidence interval [CI], 1.0-2.5, *P* = 0.036). The risk increase was significant for basal cell carcinoma (HR, 1.8, 95% CI, 1.1–2.9, *P* = 0.012), whereas there was a trend for cutaneous squamous cell carcinoma, (HR, 1.9, 95% CI, 0.9–4.1, *P* = 0.086) and cutaneous melanoma (HR, 2.4, 95% CI, 0.9–6.2, *P* = 0.083). Standardized incidence ratio for keratinocyte cancers was 2.9 (95% CI, 2.4–3.3. The results were consistent in two subgroup analyses adjusting for use of retinoid and/or immunosuppressive drugs.

**Conclusion:**

Patients with Darier disease have an increased risk of skin cancer, particularly keratinocyte carcinoma. This risk was consistent even when known risk modifiers for keratinocyte carcinoma were excluded in sensitivity analyses. The results support previously proposed molecular links between Darier disease and skin cancer, but further investigations are needed. Additional studies are also required to develop clinical management recommendations for Darier disease. In the meantime, dermatologists should be aware of the cancer risk in these patients and remain vigilant, as detecting cancer can be challenging in hyperkeratotic and/or eroded skin.

## Introduction

Darier disease (DD, OMIM #124200) is a rare autosomal dominant hereditary skin disorder of keratinization characterized by red-brown or yellow, keratotic crusted papules with an oily and warty surface that coalesce into plaques which often fissure and ooze [1]. DD is caused by heterozygous pathogenic variants of the *ATP2A2* gene which encodes the sarcoplasmic reticulum calcium ATPase-2 (SERCA2) [2]. The disease usually presents before the age of 20 years and may worsen with increasing age [3]. *De novo* mutations of the *ATP2A2* gene are common and 40–50% of all cases occur sporadically [4, 5].

Patients with DD have an increased risk of epilepsy, mild intellectual disability, diabetes, and heart failure and the disease has thus been suggested as a multiorgan systemic condition [6, 7]. This suggestion might be plausible since SERCA2 is expressed in most tissues and intracellular calcium homeostasis is fundamental both for skin and other organs.

Nationwide registries, compared to single healthcare centers, offer a valuable opportunity to establish larger study cohorts for rare diseases, potentially uncovering new comorbidities. The Swedish version of the International Classification of Diseases 10th revision (ICD-10-SE) includes approximately 3000 detailed ICD-10 codes (i.e., including more than 4 characters). This unique coding system in Sweden enables more specific analyses in epidemiological studies particularly for genodermatoses [8]. The diagnoses in the Swedish National Patient Register (NPR) have previously shown high (85–95%) positive predictive values (PPVs) [9, 10]. While previous investigations suggest that the estimated prevalence of DD is between 1/30,000 to 1/100,000 [11, 12], our recent study using the ICD-10-SE and NPR has shown that DD is more common than previously estimated, with a prevalence of 1/12,000 in Sweden [13]. In this study we have used the same cohort and data from the Swedish Cancer Register which has very high capture rate [13].

Development of cutaneous squamous cell carcinomas (cSCC) in patients with DD has previously been suggested as a model for skin carcinogenesis as shared pathogenic and molecular mechanisms in the development of cSCC and DD have been demonstrated [14–16]. Moreover, a link between DD and basal cell carcinoma (BCC) has been suggested [17]. SERCA2 regulates desmosomal and adherens junction dynamics and defects in the ATP2A2 protein led to impaired keratinocyte differentiation and adhesion [18, 19]. Studies on SERCA2-deficient keratinocytes showed excess MAP kinase signaling, lack of epidermal integrity; weakened intercellular adhesion and defective differentiation of keratinocytes [20]. It has also been reported that the ablation of *Atp2a2*gene in mice did not replicate DD pathology but led to increased age-related keratinocyte carcinomas [21, 22]. On the other hand, loss of invasion ability through E-cadherin induces an invasive phenotype and is associated with poor diagnosis in cSCCs [23]. A molecular link between DD and cutaneous melanoma (CM) has not been extensively explored, yet endoplasmic reticulum stress, which is heightened in DD patients, has been suggested as a potential connection to the pathophysiology of melanoma [24].

Although there are pathogenic mechanisms that may suggest a link between DD and skin cancer, a robust epidemiological link in humans has not been examined. The primary aim of this retrospective registry-based cohort study was to investigate the association between DD and skin cancer.

### Materials and methods

The study was carried out with the approval of the Swedish Ethical Review Authority (approval number: 2020–06202) and Sahlgrenska University Hospital (SUH). The study consisted of two stages: a local study at the SUH and a nationwide study using the Swedish NPR.

First, the code Q82.8E of the ICD-10SE was used to identify patients with DD at SUH during a five-year period (i.e., January 1, 2016 to December 31, 2020). The DD diagnoses were verified from the medical records, including clinical description, clinical images, and histopathology. The local cohort provided a comprehensive understanding of the treatment trajectories for patients diagnosed with DD. The DD diagnoses of the local cohort were also used to validate DD diagnoses recorded in the NPR for patients visiting the SUH, as previously described [13]. The DD diagnoses in the NPR demonstrated a positive predictive value of 90.9% [13], which suggests that the DD diagnoses registered in the nationwide data are sufficiently reliable for further study.

For the nationwide study, a cohort of patients with DD was generated using the NPR and the same ICD-code as above during the time period of January 1, 2001 to December 31, 2020. The NPR, hosted by the Swedish National Board of Health and Welfare, collects information on all inpatient and outpatient visits and uses the ICD-10-SE to register diagnoses. The diagnoses made in hospitals are automatically transferred from hospitals to the NPR. To each patient with DD, ten age-, sex- and municipality-matched controls were randomly drawn from the Swedish population. To investigate the association between DD and the risk for skin cancer, all patients were cross referenced with Swedish Cancer Registry. Data from the Swedish Cancer Registry was available until December 31, 2019.

Standardized incidence ratios (SIRs) were computed as the ratio of observed and expected cancers during the follow-up. The follow-up time started at the index date of each individual (the first diagnosis of DD in 2001–2020) or the analysis start date, whichever occurred the latest. The follow-up time ended at the end of study period (i.e., December 31, 2019), death or emigration. The analyses spanned 2001–2019 except for analyses involving BCC, which were analyzed over 2004–2019 due to the establishment of the BCC registry in 2004. The sum of follow-up time was stratified by calendar year, sex and 5-year age group, and the numbers of expected cancers were computed based on the cancer incidence rates of the Swedish general population in each stratum. The 95% confidence intervals (CIs) of the estimates were based on the Poisson distribution. CM was defined with the ICD-7 code 190, and SCC with the pathological-anatomical diagnosis of squamous carcinoma plus ICD-7 code 191 or 140. BCCs were obtained from the registry described above. Keratinocyte carcinoma was defined as either SCC or BCC. For the SIR analyses, only tumors included in the Swedish official cancer statistics were analyzed, i.e., in situ carcinomas were excluded.

The cumulative risk for being diagnosed with a skin cancer was computed over 2001–2019 using the Nelson-Aalen method. The different types of skin cancers were defined as in the SIR analysis. The individuals with DD entered the analysis at their index date. Death was included as a competing risk, and age was used as the underlying time scale.

To calculate the relative risk of being diagnosed with a skin cancer, a 5-year washout period was applied and only patients with index date of DD recorded between January 1, 2006, and December 31, 2015, were included in the analysis. All individuals needed to be cancer free for the respective cancer type at the index dates and were followed up to whatever came first of emigration, first event, death, or end of follow-up period (December 31, 2019). Kaplan–Meier plots covered the time from the index date to the first diagnosis of the respective type of skin cancer: BCC, cSCC (including cutaneous squamous cell carcinoma in situ), keratinocyte carcinoma (i.e., BCC and cSCC combined) and CM (including melanoma in situ). Hazard Ratios (HRs) were calculated using Cox’s proportional-hazards regression. All *P*‐values were two sided, and *P* < 0.05 was considered as statistically significant.

Since both retinoid treatment options (which is a common treatment for patients with DD) and immunosuppressive drugs may modify the risk for skin cancer, we conducted sensitivity analyses. In these analyses, individuals with use of immunosuppressive drugs and/or retinoids were excluded from the HR analyses. Use of vitamin A derivatives and immunosuppressive medications was retrieved from the Prescribed Drug Register. Retinoids were defined using the ATC codes *D05BB02* (acitretin) and *D10BA01* (isotretinoin) in the time period of January 1, 2006 to December 31, 2019. Immunosuppressive drugs were defined as any drug with ATC code starting with *L04A* in the time period of January 1, 2006 to December 31, 2019.

## Results

### Local cohort

The cohort at SUH included 13 patients with at least one registered diagnosis of DD. The diagnosis of all patients was based on typical histopathology: suprabasal fissuring and dyskeratotic keratinocytes with pycnotic nucleus and hypereosinophilic cytoplasm surrounded by a light halo. Two of the included individuals had suspected DD, but since histopathology results were inconsistent with the diagnosis, these patients were excluded from further analyses. Two of the patients initially received a diagnosis of intertriginous eczema prior to DD. The diagnosis of DD was recorded in the patient records and sent to NPR only after the histopathological diagnosis. The most frequently considered differential diagnoses in the in the referrals to pathology included Hailey-Hailey disease, seborrheic dermatitis and transient acantholytic dermatitis.

**Fig. 1:**
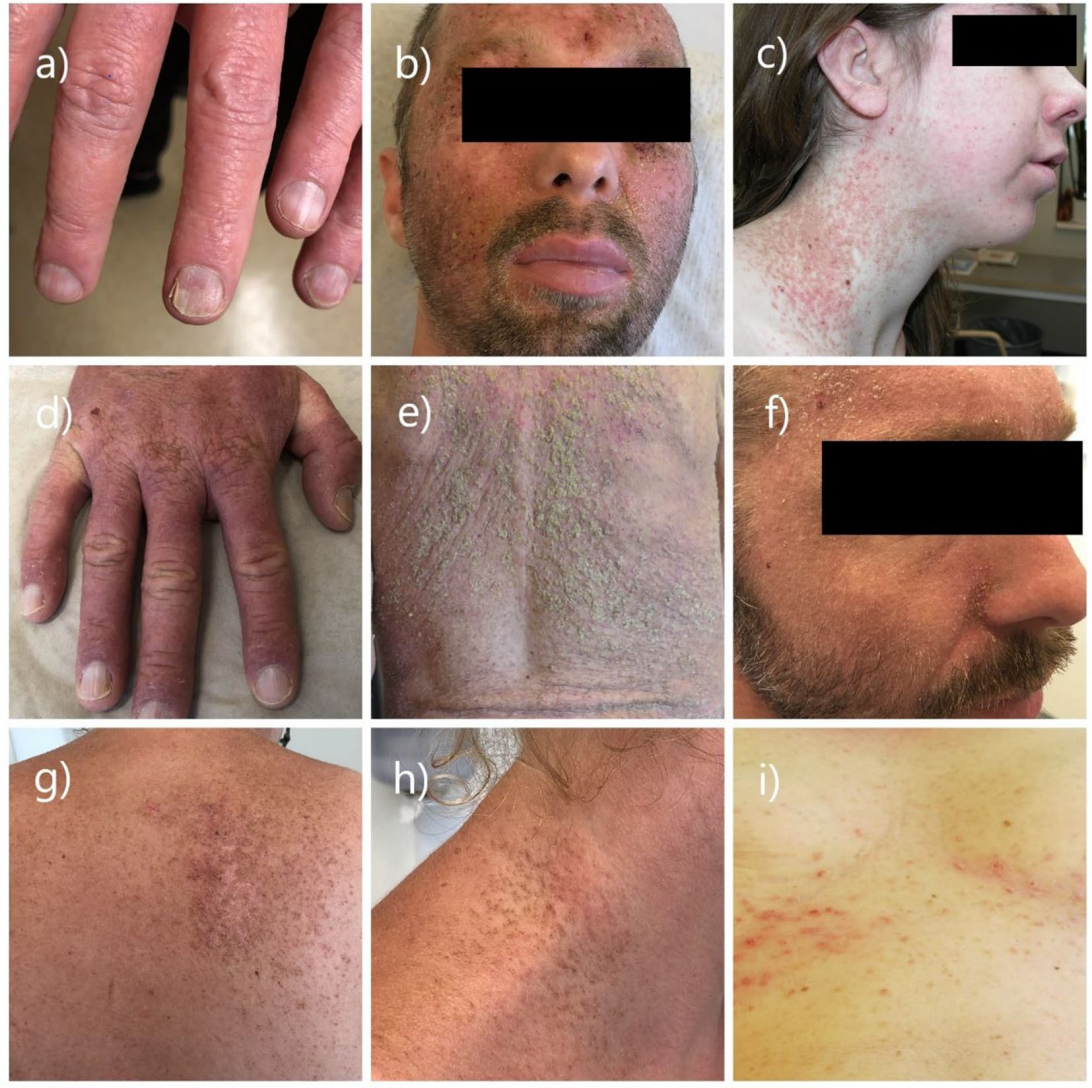
Patients with Darier diseases **a**) Typical V-shaped notch at the free margin of the nail on a patient with Darier disease, **b**) Patient with Darier disease with acute worsening in his face due to eczema herpeticum, **c**) Red-scaly itching patches in the neck and face area on a patient with Darier disease, **d**) Red thick patches at the dorsal side of hand with red-white stripes on nails, **e**) Yellowish larger warty lesions covering the back of a patient with Darier disease, **f**) Oily crusted lesions on face, similar to seborrhoeic dermatitis, **g**-**h**) Small brown papules with a firm, harsh sandpaper-like texture on the back and neck of a patient with Darier disease, **i**) Patient with Darier disease who was initially misdiagnosed as having intertriginous eczema. *The patients have provided written informed consent for the publication of their photos*

Nine (73%) of the included patients were females and two (27%) were males. The age of the included patients ranged from 32 to 76 years (median age 52 years). Most patients received their first diagnosis during or after adolescence at an average age of 20 years. Overall, 6 of the 11 patients stated that at least one first degree relative had DD. The comorbidities of patients with DD included schizophrenia, hypothyroidism (three patients), heart failure and type I diabetes. Review of the patients’ medical records illustrated that all patients were using moisturizers and a prescribed combination of topical corticosteroids/antibiotic creams. Seven patients had been prescribed oral acitretin with daily doses ranging from 10 to 40 mg. All 11 patients had their diagnosis histopathologically confirmed. Most patients exhibited considerable variation in their disease. Exposure to heat and sun were most often reported as disease triggers. The use of oral antibiotics due to secondary infections was frequent, and oral antivirals were prescribed to a patient with the diagnosis of eczema herpeticum (Fig. 1).

### National cohort

The national cohort included 770 patients (461 [60%] females and 309 [40%] males) with at least one diagnosis of DD in the time period of 2001–2020. The SIRs for skin cancers were computed over years 2001–2019 with 741 patients (448 [60.5%] females and 293 [39.5%] males). Among these patients, the mean age at the index date was 44.5 years (Table 1). The SIR for SCC was 3.8 (95% CI, 2.4–5.8), and the SIR for keratinocyte carcinomas overall was 2.88 (95% CI 2.4–3.4) (Table 2).

**Table 1:** Characteristics of the cohort used in standardized incidence ratio (SIR) and cumulative risk analyses between 2001–2019

Variable	Patients	Controls
**Number of patients**		
All patients, n	741	7405
Male, n (%)	293 (39.5)	2928 (39.5)
Female, n (%)	448 (60.5)	4477 (60.5)
**Age at entry**		
Age at entry, mean (SD)	44.5 (20.2)	44.5 (20.2)
Age at entry 0–20, n (%)	104 (14)	1040 (14)
Age at entry 20–40, n (%)	198 (26.7)	1980 (26.7)
Age at entry 40–60, n (%)	254 (34.3)	2536 (34.2)
Age at entry 60–80, n (%)	159 (21.5)	1590 (21.5)
Age at entry 80+, n (%)	26 (3.5)	259 (3.5)
**Follow up time**		
Follow-up time, sum	7205.2	73433.4
Follow-up time, mean (SD)	9.7 (5.6)	9.9 (5.6)

**Table 2:** Observed and expected numbers of skin cancers and standardized incidence ratios (SIRs) with 95% confidence intervals (CIs) among patients with Darier disease

	Individuals with cancer	Follow-up time (person-years)	Observed cancers	Expected cancers	SIR	95% CI	*P*
**Keratinocyte carcinomas (2004–2019)** ^**a**^							
All patients	58	6919	136	47.17	2.88	2.43 to 3.40	< 0.001
Males	28	2497	60	20.01	3.00	2.30 to 3.82	< 0.001
Females	30	4422	76	27.16	2.80	2.22 to 3.48	< 0.001
**Basal cell carcinomas (2004–2019)** ^**a**^							
All patients	53	6919	117	42.03	2.78	2.31 to 3.32	< 0.001
Males	24	2497	50	17.18	2.91	2.18 to 3.79	< 0.001
Females	29	4422	67	24.86	2.70	2.10 to 3.39	< 0.001
**Squamous cell carcinomas (2001–2019)** ^**b**^							
All patients	11	7205	20	5.20	3.84	2.40 to 5.78	< 0.001
Males	8	2607	11	2.88	3.82	1.98 to 6.54	< 0.001
Females	3	4598	9	2.33	3.87	1.86 to 6.98	< 0.001
**Cutaneous melanoma (2001–2019)** ^**b**^							
All patients	5	7205	5	3.39	1.47	0.53 to 3.17	0.385
Males	0	2607	0	1.34	0	-	-
Females	5	4598	5	2.05	2.44	0.87 to 5.24	0.046

^a^ among 733 individuals with DD (288 males and 445 females)

^b^ among 741 individuals with DD (293 males and 448 females)

The cumulative risk for any non-BCC skin cancer by 60 years of age was 3% (95% CI, 1–8%) among patients with DD and 1% (95% CI, 1–2%) among controls. By 80 years of age, the corresponding numbers were 10% (95% CI, 6–16%) and 5% (95% CI, 4–7%). The cumulative incidence of BCC in patients with DD was about three times that of the controls by the age of 60 years (13%, 95% CI 9–20% vs. 4%, 95% CI 3–5%), and a similar ratio remained by age 80.

**Fig. 2:**
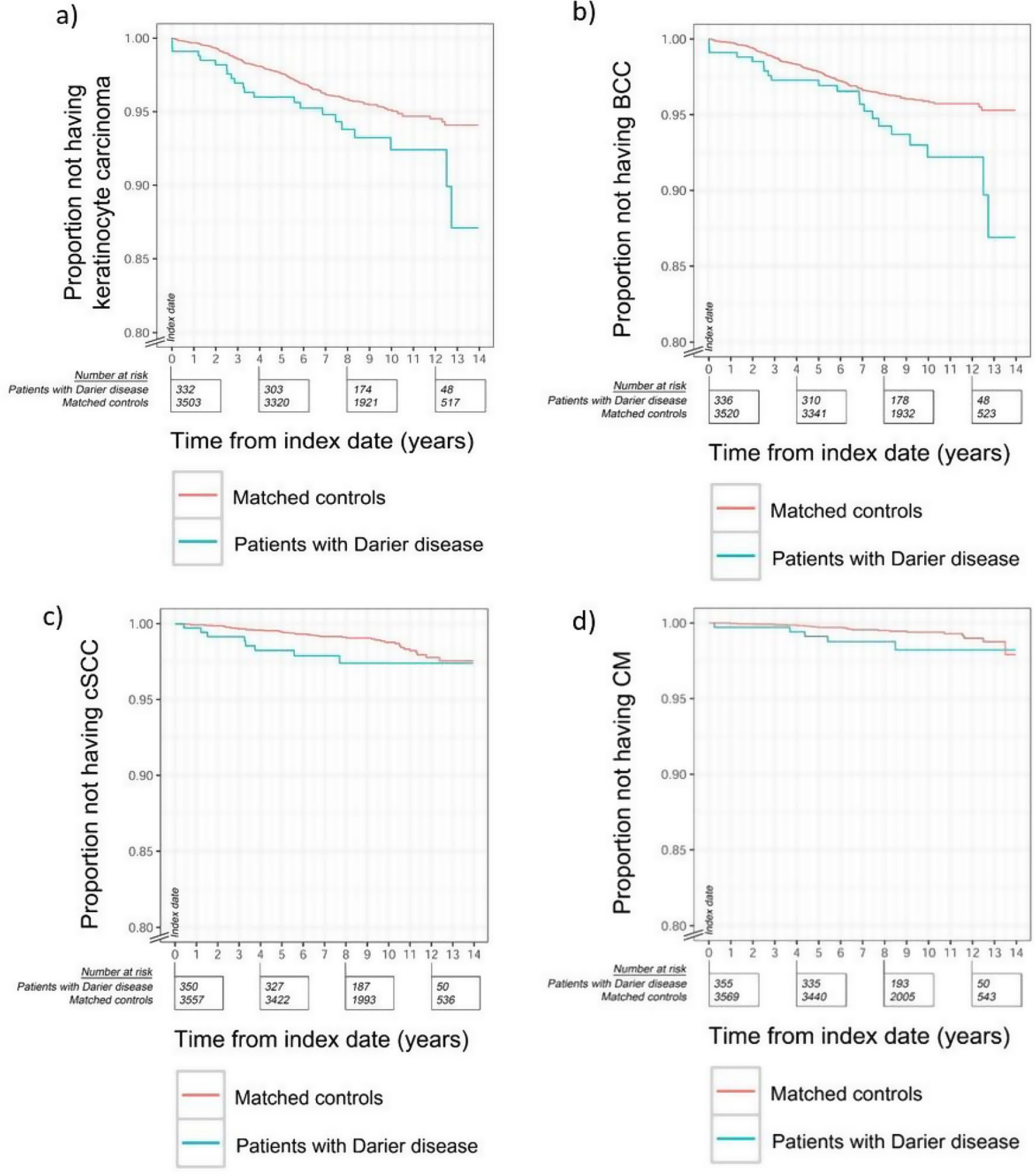
Kaplan–Meier curves showing risk for the respective cancer type. Risk of keratinocyte cancer (i.e., including both cSCC and BCC. (**a**), BCC (**b**) cSCC (**c**) and CM (**d**) among 357 patients with Darier disease and 3581 controls. The index date was in the time period of January 1, 2006 to December 31, 2015. All individuals (patients with Darier disease and controls) had no history of keratinocyte carcinoma at the index dates and were followed up to whatever came first of emigration, keratinocyte carcinoma (event), death, or end of follow-up period (December 31, 2019)

The analyses of the HR of first cancers included 357 patients (209 [58.5%] females and 148 [41.5%] males) (Table 3). The relative risk of being diagnosed with a keratinocyte carcinoma (i.e., BCC or cSCC) of DD patients was 1.6 (95% CI, 1.0-2.5, *P* = 0.036), while for BCC it was 1.8 (95% CI, 1.1–2.9, *P* = 0.012). The point estimates for cSCC (HR, 1.9, 95% CI, 0.9–4.1, *P* = 0.086) and CM (HR, 2.4, 95% CI, 0.9–6.2, *P* = 0.083) also suggested increased relative risk, yet the differences were not statistically significant (Fig. 2).

**Table 3:** Characteristics of the source cohort used in HR and sensitivity analyses for medications

Age					Ever-use of		
					Retinoids^a^		Immunosuppressive drugs^b^
*n* (%)		Median (IQR)	Mean (95% CI)	*n* (%)^c^		*n* (%)^c^	
**Patients with Darier disease**	357 (100)	48.2 (31.0-62.7)	46.4 (44.2–48.6)	107 (30.0)		24 (6.7)	
Male	148 (41.5)	47.2 (26.3–64.1)	45.5 (41.7–49.4)	44 (29.7)		5 (3.4)	
Female	209 (58.5)	48.5 (35.3–62.3)	47.1 (44.4–49.7)	63 (30.1)		19 (9.1)	
**Controls**	3581 (100)	48.2 (30.0-62.7)	46.4 (45.7–47.1)	24 (0.67)		101 (2.8)	
Male	1488 (41.6)	48.3 (26.3–64.1)	45.8 (44.6–47.0)	11 (0.74)		30 (2.0)	
Female	2093 (58.4)	48.2 (34.3–62.3)	46.8 (45.9–47.6)	13 (0.62)		71 (3.4)	

^a^ Immunosuppressive drugs were defined as any drug with ATC code starting with *L04A* in the time period of January 1, 2006 to December 31, 2019

^b^ Retinoids were defined as any drug starting with ATC codes *D05BB02* (acitretin) and/or *D10BA01* (isotretinoin) in the time period of January 1, 2006 to December 31, 2019

^c^ Proportion of individuals exposed in the respective group. ATC, Anatomical Therapeutic Chemical; CI, confidence interval; DD, Darier disease; IQR, interquartile range

**Fig. 3:**
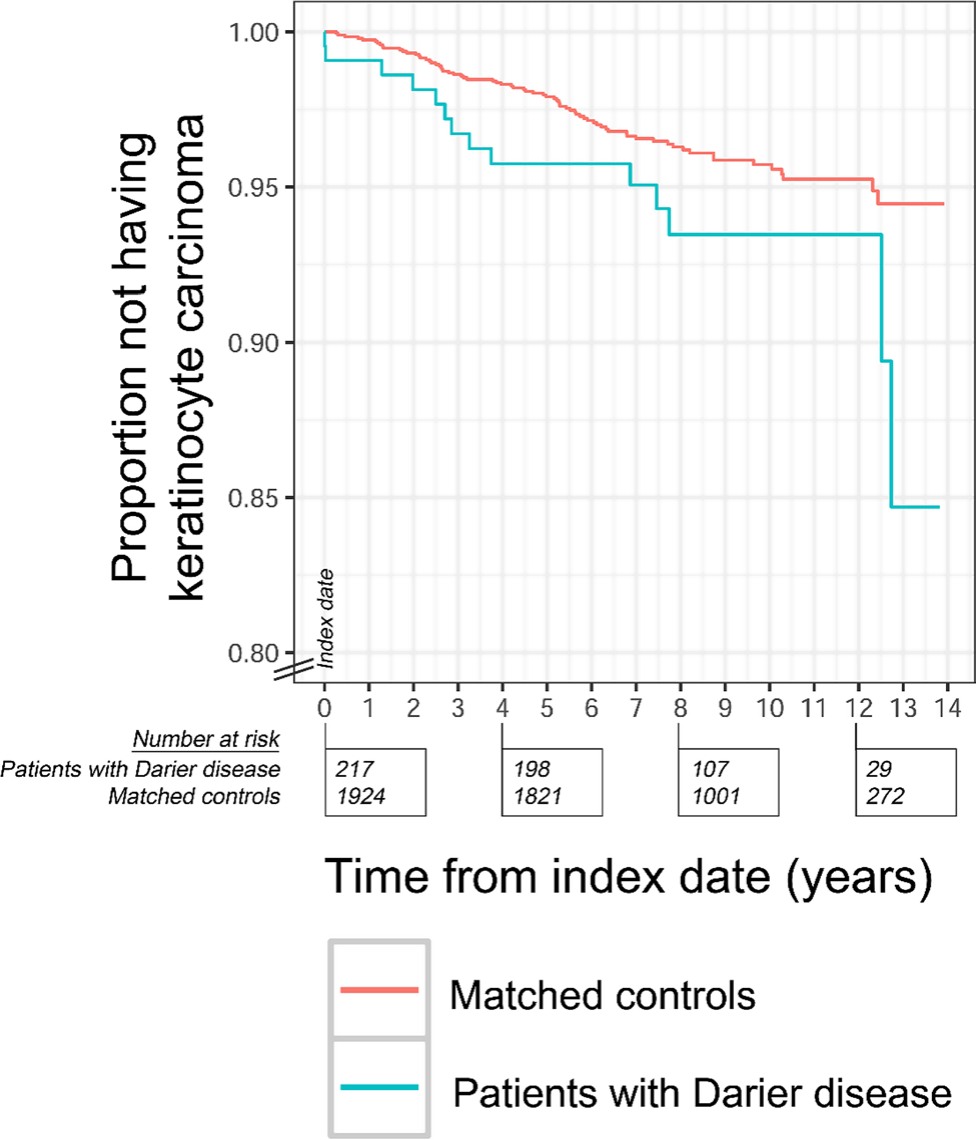
Risk of keratinocyte carcinoma among patients with Darier disease without use of retinoids or immunosuppressive drugs and their corresponding controls Kaplan–Meier curves showing cases of keratinocyte carcinoma (both cSCC and BCC). The index date was in the time period of January 1, 2006 to December 31, 2015. All individuals (patients with Darier disease and controls) had no history of keratinocyte carcinoma at the index dates and were followed up to whatever came first of emigration, keratinocyte carcinoma (event), death, or end of follow-up period (December 31, 2019)

The use of retinoids and immunosuppressive drugs was more common among patients with DD compared to controls (30.0% vs. 0.67%, *P* < 0.001, and 6.7% vs. 2.8%, *P* < 0.001, respectively) (Table 3). The HRs were computed after the exclusion of patients with use of immunosuppressive drugs and/or retinoids (Supplemental material). For keratinocyte carcinoma, the exclusion of all patients with use of retinoids yielded a HR of 1.9 (95% CI, 1.1–3.3, *P* = 0.016). The exclusion of all patients with use of immunosuppressive drugs led to a HR of 1.6 (95% CI, 1.0-2.7, *P* = 0.048). Finally, when all patients with either immunosuppressive drugs or retinoids were excluded, the HR for keratinocyte carcinoma was 1.8 (95% CI, 1.0-3.1, *P* = 0.051) (Fig. 3).

## Discussion

Prior research has not confirmed a link between DD and skin cancer. In this study we found a significant association, indicating an elevated risk of skin cancer in patients with DD.

Conducting research on rare diseases presents challenges due to the limited number of patients available. Also collecting data to determine whether a disease has an association with an increased risk of cancer poses difficulties because prospective design requires long follow-up periods. Notably, our national cohort comprises a large number of patients with DD and benefits from a long observation time.

Although the local cohort from SUH was small, it provided us with important information regarding the diagnostic process of DD and the validity of the diagnoses. The sex distribution mirrored that of the national cohort. Additionally, the comorbidities were consistent with those previously published [25].

The results of the present study showed that both the SIR and HR for BCC were significantly increased in patients with DD, and cancer incidence increased at a slightly younger age in patients with DD compared to the controls. The results also suggest an increased incidence of cSCC and CM. Previous knowledge on the association between DD and cancer is based on case reports which have shown BCCs, cSCCs and an amelanotic CM arising on affected skin of DD patients [15, 17, 26, 27]. Non-cutaneous malignancies associated with DD have also been reported including Hodgkin’s disease [28]. An association between DD and cancer is plausible since same pathogenic mechanisms including disturbances in cell-cell adhesion may play a role in SCC and DD. Decreased SERCA activity could cause increased entry of calcium which may affect cell proliferation and resistance to apoptosis [29]. E-cadherin mediates cell-cell adhesion, and it has been shown in DD, that E-cadherin expression is absent or poor at the periphery of the acantholytic cells which leads to proteolytic destruction [30, 31]. Furthermore, this may disturb cell adhesion [32]. Decreased epidermal cell adhesion in severe DD leads to chronic epidermal wounding and could contribute to carcinogenesis [33]. This hypothesis implies that the long-lasting erosions in DD may create an environment conducive to the development of carcinoma [31].

One strength of our investigation is the analysis of retinoid use which is common in this patient group. It is known that the use of acitretin reduces the risk of skin cancer [34]. Slightly higher point estimates in the analysis excluding patients with retinoids suggested that retinoids might be protective, but our material is insufficient to draw conclusions. On the other hand, individuals that are immunocompromised, either due to disease or medication, have an increased risk for malignant conversion [35]. However, the present sensitivity analysis excluding individuals with pharmacological immunosuppression yielded an unchanged HR estimate for keratinocyte carcinoma.

This investigation was limited by its retrospective design and small sample size at the local cohort. The diagnoses in the national cohort could not be verified in the medical records, yet the PPV for DD in the NPR has previously been found to be high. We therefore believe that the national cohort is sufficiently reliable for cancer studies. DD affects all ethnicities; our investigation was conducted in Sweden where most citizens have skin types ranging from 1 to 3.

No data on exposure to ultraviolet light was available and the association observed might be inflated due to surveillance bias (i.e., more frequent visits to a dermatologist among patients with DD). On the other hand, UV and warm weather are known exacerbating factors for DD and many patients avoid sun exposure.

## Conclusion

To summarize, our results suggest an increased risk for skin cancer, and particularly keratinocyte carcinoma among patients with DD. The findings were consistent in sensitivity analyses, where known risk modifiers for keratinocyte carcinoma were excluded. The results also align with the previously proposed molecular links between DD and cSCC but warrant further investigations in other populations with more diverse skin types. More studies are also needed in order to suggest recommendations for clinical management of DD. Meanwhile, it is important that dermatologists treating these patients are aware of cancer risk and remain alert since detecting cancer can be difficult in hyperkeratotic and/or eroded skin.

## Data Availability

Data are available upon reasonable request for researchers though data access is restricted. Please contact the National Board of Social Affairs and Health, https://www.socialstyrelsen.se/en/ for permission.

## Abbreviations

DD: Darier Disease
CI: Confidence Interval
HR: Hazard Ratio
BCC: Basal Cell Carcinoma
cSCC: cutaneous Squamous Cell Carcinoma
CM: Cutaneous Melanoma
SIRs: Standardized Incidence Ratios
SERCA2: Sarcoplasmic Reticulum Calcium ATPase-2
ICD-10-SE: Swedish version of the International Classification of Diseases 10th revision
SUH: Sahlgrenska University Hospital
NPR: Swedish National Patient Register
PPVs: Positive Predictive Values
OMIM: Online Mendelian Inheritance in Man
